# Successful Treatment of Antiepileptic Drug-Induced DRESS Syndrome with Pulse Methylprednisolone

**DOI:** 10.1155/2013/928910

**Published:** 2013-04-18

**Authors:** Celebi Kocaoglu, Ceyda Cilasun, Ece Selma Solak, Gulcan S. Kurtipek, Sukru Arslan

**Affiliations:** ^1^Department of Pediatrics, Konya Education and Research Hospital, Meram, 42090 Konya, Turkey; ^2^Department of Dermatology, Konya Education and Research Hospital, 42090 Konya, Turkey; ^3^Department of Pediatric Rheumatology, Konya Education and Research Hospital, 42090 Konya, Turkey

## Abstract

Drug reaction with eosinophilia and systemic symptoms (DRESS) syndrome is a rare but potentially life-threatening syndrome characterized by skin rash, fever, lymph node enlargement, and involvement of internal organs. It is most commonly induced by aromatic anticonvulsants and antibiotics. Nonaromatic anticonvulsants are rarely encountered as the causes of DRESS syndrome. In the present report, three discrete cases with DRESS syndrome developing due to three antiepileptic drugs, including valproic acid (nonaromatic), carbamazepine (aromatic), and lamotrigine (aromatic), and their treatment modalities were aimed to be discussed in light of the literature. To the best of our knowledge, our cases are the first children to be treated with pulse methylprednisolone in the literature.

## 1. Introduction

DRESS syndrome reflects a serious hypersensitivity reaction, especially to antiepileptic drugs. Clinical features include cutaneous eruption, fever, multiple peripheral lymphadenopathies, and potentially life-threatening damage of one or more organs, such as hepatitis, nephritis, or myocarditis. Skin rash, suggestive of DRESS syndrome, includes maculopapular rash or generalized erythematous rash, usually associated with facial edema [1, 2]. Reversion of systemic manifestations is very slow, ranging between 1 and 6 months [3].

Liver is the most frequently affected internal organ [1, 4, 5]. Other systemic involvements like interstitial nephritis, encephalitis, aseptic meningitis, myocarditis, interstitial pneumonitis, or vasculitis may also be seen. Pathogenesis of DRESS remains unclear. Different mechanisms, like detoxification defects causing reactive metabolite formation and subsequent immunological reactions, slow acetylation, and reactivation of human herpesviruses, were implicated in its development [2]. DRESS syndrome is most commonly induced by aromatic anticonvulsants and antibiotics. Nonaromatic anticonvulsants are rarely encountered as the causes of DRESS syndrome [2, 3, 6, 7].

In this report, three discrete cases with DRESS syndrome developing due to three antiepileptic drugs, including valproic acid ((VPA), nonaromatic), carbamazepine (CBZ, aromatic), and lamotrigine ((LMT), aromatic), and their treatment modalities were aimed to be discussed in light of the literature. To the best of our knowledge, our cases are the first children to be treated with pulse methylprednisolone in the literature.

## 2. Case Presentations

### 2.1. Case 1

A 12-year-old boy was admitted to the emergency department with the complaints of high grade fever, weakness, and generalised erythematous eruption. Epilepsy was present in history. VPA treatment had been started three weeks before admission. No history of animal/insect bites was detected.

**Figure 1:**
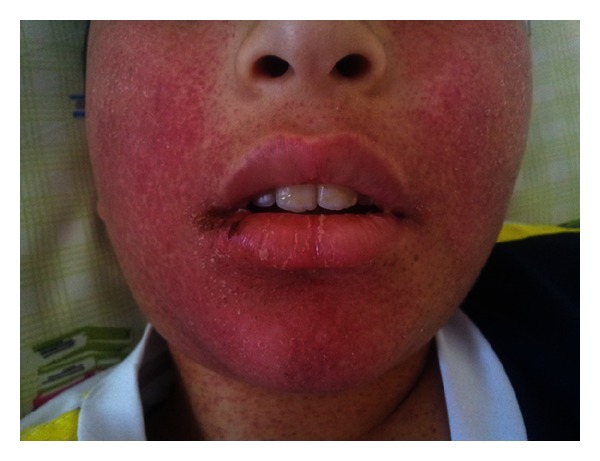
Generalised maculopapular rash, partly confluent to plaques on face.

**Figure 2:**
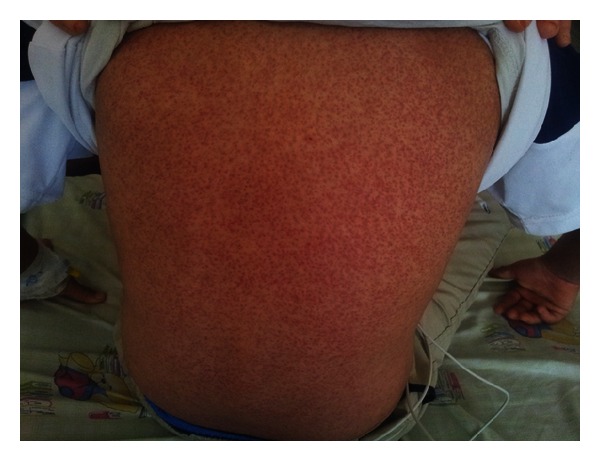
Generalised maculopapular rash on back.

On examination, he was awake and alert and displayed no acute distress. Vital signs were temperature, 39°C, pulse rate, 88 bpm, respiration, 18 breaths/min, and blood pressure, 110/75 mmHg. Skin showed generalised maculopapular rash, partly confluent to plaques on body, face, and back (Figures 1 and 2). Furthermore, he had facial edema and hepatomegaly without stigmata of chronic liver disease. Physical examination revealed no lymphadenopathy.

Laboratory findings revealed hemoglobin (12.2 g/dL) and leucocyte (3.07 × 10^3^/mm^3^) counts with a differential of 20% neutrophils, 64% lymphocytes (7% consisted of atypical lymphocytes with basophilic cytoplasm including vacuoles and larger than normal), and 16% eosinophils. Aspartate aminotransferase, alanine aminotransferase, alkaline phosphatase, and gamma-glutamyl transferase were elevated at 216, 235, 405, and 481 IU/L, respectively. C-reactive protein was elevated at 30.2 mg/dL (normal range < 5 mg/L). Erythrocyte sedimentation rate was 50 mm/h (normal range < 20 mm/h). Serum amylase, urea, and electrolytes were normal. Blood and throat cultures were negative. Virological examinations for hepatitis A, B, and C, the Epstein-Barr virus, parvovirus B-19, human herpesvirus type 6, and cytomegalovirus were negative. Antistreptolysin O, anti-double-stranded DNA, and ANA profiles were negative. Lactate dehydrogenase level was elevated to 936 U/L (normal range 120–330 U/L). VPA level was 97.5 *μ*g/mL (normal range 50–100 *μ*g/mL). Ultrasonography revealed hepatosplenomegaly.

The patient was diagnosed with probable case of DRESS syndrome based on clinical and laboratory findings (Table 1). Previous medication, VPA, was abruptly discontinued and replaced by levetiracetam. Antihistamine therapy was initiated to be administered. In the following days, laboratory tests showed further elevation of hepatic enzymes. Intravenous immunoglobulin was given as a dose of 400 mg/kg/day for 4 days. Owing to unresponsiveness, pulse methylprednisolone was given at a dose of 30 mg/kg (max 1 g/day) for 3 days, and a good response to the treatment was observed. Fever and rash disappeared with resolving of facial edema in one week. Leucocyte count increased from 3.07 × 10^3^/mm^3^ to 10.2 × 10^3^/mm^3^. He was discharged from the hospital in good condition with oral prednisolone treatment at a dose of 1 mg/kg/day. After two weeks, all symptoms completely resolved, laboratory tests were normal, and oral prednisolone was discontinued.

### 2.2. Case 2

A 9-year-old girl was admitted to the clinic with the complaint of fever and widespread skin rash. The history revealed that the girl had been taking VPA for five years for the treatment of epilepsy, and CBZ had been added one month before the admission. With the complaints of high fever and skin rash 25 days after initiation of CBZ treatment, the girl had been treated for the diagnosis of scarlet fever by her family doctor. Due to the continuation of complaints, she was referred to our hospital. 

On examination, physical findings were as follows: temperature, 39°C, pulse rate, 88 bpm, respiration, 24 breaths/min, and blood pressure, 105/70 mmHg. Skin rash tended to be confluent and was widespread on body. Bilateral cervical and suboccipital enlarged lymph nodes and hepatomegaly were determined. 

Laboratory findings revealed hemoglobin (11.2 g/dL) and leucocyte counts (9.3 × 10^3^/mm^3^) with a differential of 59% lymphocytes (9% were composed of atypical lymphocytes), 24% neutrophils, and 15% monocyte. Eosinophils number was 0.77 × 10^3^/mm^3^. Lymphocyte level (5.5 × 10^3^/mm^3^) was above the laboratory limits (0.9–3.2 × 10^3^/mm^3^). Aspartate aminotransferase, alanine aminotransferase, alkaline phosphatase, and gamma-glutamyl transferase were elevated at 323, 216, 280, and 454 IU/L, respectively. C-reactive protein was 3.19 mg/dL (normal range < 5 mg/L). Erythrocyte sedimentation rate was 9 mm/h. Blood and throat cultures were negative. Virological examinations for hepatitis A, B, and C, the Epstein-Barr virus, parvovirus B-19, human herpes virus type 6, and cytomegalovirus were negative. Antistreptolysin O, antidouble-stranded DNA, and ANA profiles were negative. Lactate dehydrogenase level was elevated to 784 U/L (normal range 120–330 U/L). VPA and CBZ levels were 77.5 *µ*g/mL and 8 *µ*g/mL, respectively. Ultrasonography revealed hepatosplenomegaly.

**Figure 3:**
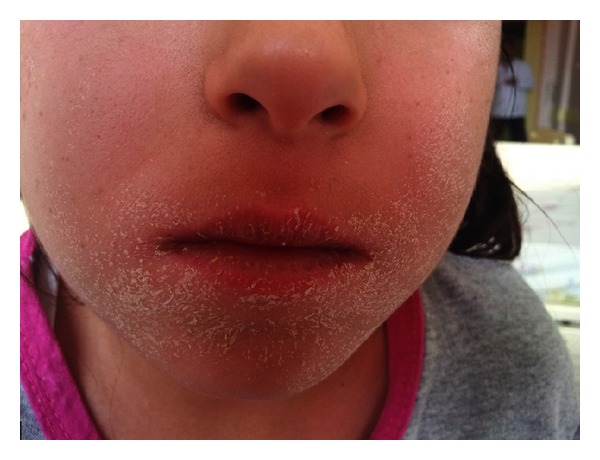
During the improvement process, desquamation on face.

The patient was diagnosed with definite case of DRESS syndrome based on clinical and laboratory findings. CBZ was abruptly discontinued, while VPA treatment was continued. Pulse methylprednisolone was given at a dose of 30 mg/kg (max 1 g/day) for 3 days. Fever and rash disappeared with resolving of facial edema in 8 days. While improving, marked peroral desquamation was remarkable (Figure 3). The case was discharged from the hospital with oral prednisolone treatment at a dose of 1 mg/kg/day. After two weeks, all symptoms completely resolved, laboratory tests were normal, and oral prednisolone was discontinued.

### 2.3. Case 3

A 6-year-old boy was admitted to the emergency with the complaints of high fever and generalised erythematous eruption. Two weeks earlier, LMT treatment had been started to the patient who was followed with the diagnosis of mental motor retardation and ventriculoperitoneal shunt, and on the use of VPA due to epilepsy. On day 10 of LMT treatment, maculopapular rash on whole body, mainly, on the face and upper extremities, was witnessed.

On examination, physical findings were as follows: temperature, 38.7°C, pulse rate, 108 bpm, respiration, 34 breaths/min, and blood pressure, 90/60 mmHg. Maculopapular skin rash tended to be confluent and was widespread on body. Bilateral enlarged cervical lymph nodes were determined.

Laboratory findings revealed hemoglobin (12.8 g/dL) and leucocyte (5.2 × 10^3^/mm^3^) counts with a differential of 37% lymphocytes (6% consisted of atypical lymphocytes), 56% neutrophils, and 5% monocyte. Eosinophils number was 1.38 × 10^3^/mm^3^. Platelet level (194 × 10^3^/mm^3^) was below the laboratory limits (217–497 × 10^3^/mm^3^). Aspartate aminotransferase, alanine aminotransferase, alkaline phosphatase, and gamma-glutamyl transferase were 19, 20, 230, and 18 IU/L, respectively. C-reactive protein was 9.27 mg/dL (normal range < 5 mg/L). Erythrocyte sedimentation rate was 6 mm/h. Antistreptolysin O titer was normal. While valproic acid level was 87.5 *µ*g/mL, level of lamotrigine could not be investigated.

The patient was diagnosed with probable case of DRESS syndrome based on clinical and laboratory findings. While LMT was abruptly discontinued, VPA treatment was continued. Owing to the lack of internal organ involvement, no steroid therapy was considered for the patient. Hydroxyzine and cetirizine were administered. Following 3-day hospitalization, the case was discharged to be followed up in outpatient clinic. On tenth-day hospital visit, the case was seen to have no complaints, and laboratory findings were normal.

## 3. Discussion

DRESS syndrome is a rather distinct severe adverse drug reaction characterised by skin rash, fever, lymph node enlargement, and single or multiple organ involvement. Cutaneous lesions can range from erythematous papules to plaques, pustules, and eczematous lesions [8]. Systemic involvement includes hepatitis, interstitial nephritis, or pneumonitis. In the literature, hepatitis is reported to be common and occur in up to 90% of cases, as congruent with our cases. Renal (9%) or pulmonary involvement (5%) is less commonly described [2]. 

DRESS syndrome usually manifests itself within 1–8 weeks after drug therapy [9]. In our cases, fever and rash developed 2 to 3.5 weeks after exposure to drug, and eruptions were characterized by maculopapular rash on face, trunk, and all extremities. As reported that liver transaminases are increased in 50% of cases in the literature [10], increased levels of liver transaminases were detected on admission in our Cases 1 and 2 as well. In addition, fever and eosinophilia were also present in all cases. 

DRESS syndrome mimics many other diseases. In its differential diagnosis, septicaemia, autoimmune diseases including vasculitides, tick-borne diseases, and other diseases like viral hepatitis should be taken into consideration [11, 12]. These conditions can be excluded in all cases of suspected DRESS through either relevant history or serology. Our Case 2 was also tried to be treated as a result of misdiagnosis of scarlet fever. Existence of herpesviruses, particularly herpes virus type 6, is suggestive of diagnosis and may be a cofactor in the pathogenesis of DRESS syndrome [13]. Infectious diseases were ruled out through viral and bacterial examinations, and connective tissue disorders were ruled out through negative antidouble-stranded DNA and ANA profiles. RegiSCAR score, diagnostic criteria developed by The European Registry of Severe Cutaneous Adverse Reactions to Drugs and Collection of Biological Samples to assist the diagnosis of drug hypersensitivity syndrome, was determined as 5, 6, and 4 in Cases 1, 2, and 3, respectively [14].

The first modality in the treatment of DRESS syndrome is to discontinue the causative drug. Patients with DRESS, although optimum treatment remains controversial, are usually treated with corticosteroid [1, 2]. In individual cases, treatments with corticosteroids and intravenous immunoglobulin are reported to be effective; however, no controlled trials of such therapies are present. Mean recovery time is 6.4 ± 9.4 weeks [2]. As different from the regimes including conventional oral doses reported in previous studies, parenteral pulse corticosteroid therapy used in our modality is considered to be more successful because of both a more rapidly favorable clinical course and returning of liver tests to normal in shorter period. Treatments of pulse methylprednisolone at a dose of 30 mg/kg (max 1 g/day) and oral methylprednisolone at a dose of 1 mg/kg were successfully carried out in Cases 1 and 2. As a result of treatment of pulse methylprednisolone plus oral methylprednisolone, healing process was seen to take shorter time, compared with those reported in the literature [2, 3]. 

Consequently, while evaluating skin rash, fever, systemic involvement, and eosinophilia, healthcare professionals should be alerted to DRESS syndrome, and in the differential diagnosis, scarlet fever should also be kept in mind. To the best of our knowledge, these cases are the first to be treated with pulse methylprednisolone. In such cases, prompt recognition and withdrawal of the causative drug are essential. Treatment with pulse methylprednisolone may be beneficial in the treatment of cases with DRESS syndrome, especially accompanied by internal organ involvement.

## Figures and Tables

**Table 1:** Scoring system for classifying the cases of DRESS as possible, probable, or definite.

Scores	Case 1	Case 2	Case 3
Fever ≥ 38.5°C	0	0	0
Enlarged lymph nodes	0	1	1
Eosinophils 0.7–1.49 × 10^3^/mm^3^ = 1, ≥1.5× 10^3^/mm^3^ = 2	—	1	1
If leucocytes < 4 × 10^3^/mm^3^ Eosinophils, 10–19.9% = 1, ≥20% = 2	1	—	—
Atypical lymphocytes	1	1	1
Skin rash extent (>50% body surface area)	1	1	1
Skin rash suggesting DRESS	1	1	1
Organ involvement	1	1	—
Resolution ≥ 15 days	0	0	−1

Total score	5	6	4

Final score < 2, no case; final score 2-3, possible case; final score 4-5, probable case; final score > 5, definite case.
